# Early-Onset Network Hyperexcitability in Presymptomatic Alzheimer’s Disease Transgenic Mice Is Suppressed by Passive Immunization with Anti-Human APP/Aβ Antibody and by mGluR5 Blockade

**DOI:** 10.3389/fnagi.2017.00071

**Published:** 2017-03-24

**Authors:** Syed F. Kazim, Shih-Chieh Chuang, Wangfa Zhao, Robert K. S. Wong, Riccardo Bianchi, Khalid Iqbal

**Affiliations:** ^1^Robert F. Furchgott Center for Neural and Behavioral Science and Department of Physiology and Pharmacology, State University of New York (SUNY) Downstate Medical CenterBrooklyn, NY, USA; ^2^Department of Neurochemistry and SUNY Downstate/NYSIBR Center for Developmental Neuroscience, New York State Institute for Basic Research (NYSIBR)Staten Island, NY, USA; ^3^Graduate Program in Neural and Behavioral Science, SUNY Downstate Medical CenterBrooklyn, NY, USA

**Keywords:** Alzheimer’s disease, amyloid β precursor protein (APP), amyloid β (Aβ), CA3, epilepsy, hippocampus, metabotropic glutamate receptor 5 (mGluR5), seizure

## Abstract

Cortical and hippocampal network hyperexcitability appears to be an early event in Alzheimer’s disease (AD) pathogenesis, and may contribute to memory impairment. It remains unclear if network hyperexcitability precedes memory impairment in mouse models of AD and what are the underlying cellular mechanisms. We thus evaluated seizure susceptibility and hippocampal network hyperexcitability at ~3 weeks of age [prior to amyloid beta (Aβ) plaque deposition, neurofibrillary pathology, and cognitive impairment] in a triple transgenic mouse model of familial AD (3xTg-AD mouse) that harbors mutated human Aβ precursor protein (APP), tau and presenilin 1 (PS1) genes. Audiogenic seizures were elicited in a higher proportion of 3xTg-AD mice compared with wild type (WT) controls. Seizure susceptibility in 3xTg-AD mice was attenuated either by passive immunization with anti-human APP/Aβ antibody (6E10) or by blockade of metabotropic glutamate receptor 5 (mGluR5) with the selective antagonist, 2-methyl-6-(phenylethynyl)pyridine hydrochloride (MPEP). In *in vitro* hippocampal slices, suppression of synaptic inhibition with the GABA_A_ receptor antagonist, bicuculline, induced prolonged epileptiform (>1.5 s in duration) ictal-like discharges in the CA3 neuronal network in the majority of the slices from 3xTg-AD mice. In contrast, only short epileptiform (<1.5 s in duration) interictal-like discharges were observed following bicuculline application in the CA3 region of WT slices. The ictal-like activity in CA3 region of the hippocampus was significantly reduced in the 6E10-immunized compared to the saline-treated 3xTg-AD mice. MPEP acutely suppressed the ictal-like discharges in 3xTg-AD slices. Remarkably, epileptiform discharge duration positively correlated with intraneuronal human (transgenic) APP/Aβ expression in the CA3 region of the hippocampus. Our data suggest that in a mouse model of familial AD, hypersynchronous network activity underlying seizure susceptibility precedes Aβ plaque pathology and memory impairment. This early-onset network hyperexcitability can be suppressed by passive immunization with an anti-human APP/Aβ antibody and by mGluR5 blockade in 3xTg-AD mice.

## Introduction

Alzheimer’s disease (AD) is a chronic progressive neurodegenerative disorder characterized by profound cognitive deficits (Blennow et al., 2006). The hippocampus plays a key role in learning and memory (Morris et al., 1982; Burgess et al., 2002; Neves et al., 2008), and is one of the first brain regions affected by AD pathological hallmarks, i.e., amyloid beta (Aβ) plaques composed of Aβ peptide and neurofibrillary tangles composed of abnormally hyperphosphorylated tau protein (Braak and Braak, 1991, 1995, 1996; Morrison and Hof, 2002). Despite the considerable progress in deciphering the molecular pathology underlying neurodegeneration in AD over the last three decades, current understanding of the physiological basis of memory loss in AD, the most common cause of dementia, is limited.

In recent years, based primarily on critical evidence obtained from mouse models of AD, a new hypothesis has emerged that implicates the role of neuronal hyperexcitability, hypersynchronous network activity and aberrant hippocampal network rewiring in memory loss in AD (Palop et al., 2007; Palop and Mucke, 2009; Noebels, 2011; Chin and Scharfman, 2013). It is well known that AD patients are at increased risk for developing seizures and epilepsy (Friedman et al., 2012). Early-onset familial AD, caused by genetic mutations in Aβ precursor protein (APP), presenilin-1 (PS1), and presenilin-2 (PS2), is associated with a remarkable 87-fold higher seizure incidence compared to the general population (Amatniek et al., 2006; Cloyd et al., 2006). In contrast, the late-onset sporadic AD is associated with a 3-fold rise in seizure incidence (Amatniek et al., 2006; Cloyd et al., 2006). Seizures and epilepsy are particularly pronounced in families with presenilin mutations; nearly 30% of such AD patients display this comorbidity, with nearly 75% suffering from seizures and epilepsy in cases of particularly aggressive variants with early-onset AD, i.e., before age 40 (Snider et al., 2005; Larner and Doran, 2006; Jayadev et al., 2010).

AD is a risk factor for seizures, and seizures in AD are thought to be a consequence of neurodegeneration (Scarmeas et al., 2009). However, during the last decade, several mouse model studies have challenged this idea, and have suggested a different view regarding the relationship between epileptic seizures and AD, i.e., instead of being a complication of AD, epileptiform activity including both convulsive and non-convulsive seizures may contribute to cognitive impairment and disease progression in AD (Leonard and McNamara, 2007). In a landmark study, Palop et al. (2007) reported the presence of frequent epileptiform activity including spikes and sharp waves, and intermittent unprovoked seizures involving neocortex and hippocampus on video electroencephalographic (EEG) recordings in 4–7 month-old human APP mice, J20 (carrying human APP Swedish and Indiana mutations). At this age, these mice show behavioral and synaptic deficits but no obvious neuronal loss (Palop et al., 2007). These data suggested that Aβ deposition is sufficient to elicit epileptiform activity in the absence of neurodegeneration. In the same study, epileptic activity was found to lead to compensatory inhibitory remodeling of the hippocampal circuitry to counteract network activity imbalances (Palop et al., 2007). It has been proposed that both the recurrent seizure activity and compensatory homeostatic responses to this seizure activity may interfere with normal neuronal and synaptic functions essential for learning and memory (Leonard and McNamara, 2007; Palop and Mucke, 2009; Noebels, 2011; Scharfman, 2012a,b; Chin and Scharfman, 2013).

Besides J20 mice, in recent years, network hypersynchronization and epileptic activity have been documented in several other mouse models of AD. Several transgenic mice that carry human APP mutation(s) resulting in APP/Aβ overexpression display spontaneous seizures, increased pro-convulsant-induced seizure susceptibility, sharp wave discharges (SWDs) or interictal spikes (for review, Born, 2015). Interestingly, many AD transgenic mouse models carrying human APP mutation(s) display epileptiform activity and seizure susceptibility prior to amyloid plaque deposition and cognitive impairment (Del Vecchio et al., 2004; Minkeviciene et al., 2009; Westmark et al., 2010; Davis et al., 2014; Bezzina et al., 2015; Duffy et al., 2015). AD mouse models exhibit an age-dependent neuropathological process and cognitive decline. Most of the AD mouse models which harbor human APP mutation(s) and exhibit early-onset epileptiform activity and seizure susceptibility have increased intraneuronal human APP and Aβ prior to extracellular Aβ deposition and amyloid plaque formation (Oddo et al., 2003a,b; Billings et al., 2005; Lithner et al., 2011; Stargardt et al., 2015). In human AD brains, intraneuronal Aβ accumulation also precedes plaque formation (Gyure et al., 2001; Bossers et al., 2010). In AD transgenic mice, intraneuronal Aβ has been suggested to contribute to cognitive impairment prior to amyloid plaque stage (Oddo et al., 2003b; Billings et al., 2005), and aberrant network excitability may be a mechanism of this cognitive deficit.

APP, like Aβ, may also be a major contributor to network hyperexcitability in AD patients and transgenic mice. Remarkably, genetic suppression of transgenic APP in a human APP mouse model of AD has been shown to rescue hypersynchronous network activity (Born et al., 2014). Additionally, increased seizure risk has been documented in patients with APP duplication (Cabrejo et al., 2006) and in Down syndrome (DS) individuals (carrying 3 copies of the APP gene) with dementia (Menéndez, 2005).

Intraneuronal APP/Aβ may be the major culprit in the early-onset network hyperexcitability in AD which may ultimately contribute to cognitive impairment. Nonetheless, the exact mechanism of early-onset network hypersynchronization in AD, remains largely unknown. Also, if intraneuronal APP/Aβ mediate this hypersynchronous network activity, the contributory role of APP vs. Aβ and the downstream effectors remain to be elucidated. In the present study, we evaluated: (1) if the seizure susceptibility and hippocampal network hypersynchrony precede extensive neuropathology and cognitive deficit; and (2) what could be the possible cellular mechanism(s) underlying epileptic activity in a triple transgenic mouse model of familial AD. We investigated network hypersynchrony at 3 weeks of age (prior to amyloid plaque deposition, neurofibrillary pathology, and cognitive impairment) in a triple transgenic mouse model of AD (3xTg-AD) that harbors mutated human APP, tau and PS1 genes (Oddo et al., 2003b). The earliest cognitive deficits reported in 3xTg-AD mice are by 2–3 months of age (Davis et al., 2013; Stevens and Brown, 2015). However, most studies show cognitive impairment in 3xTg-AD by ~5 months of age (Oddo et al., 2003b; Billings et al., 2005). We found that 3xTg-AD mice exhibit aberrant network activity and enhanced seizure susceptibility at as early as 3 weeks of age, i.e., before the appearance of amyloid plaque and neurofibrillary pathologies, and the onset of memory impairment. Our data indicate that this early-onset network hyperexcitability in 3xTg-AD mice can be suppressed by passive immunization with an anti-human APP/Aβ antibody and by metabotropic glutamate receptor 5 (mGluR5) blockade.

## Materials and Methods

### Animals and Housing

The 3xTg-AD mice represent one of the most biologically relevant animal models described thus far as these mice replicate all histopathological and behavioral hallmarks of AD (Oddo et al., 2003b). The 3xTg-AD mice carry three AD-related genetic loci: human PS1 M146V, human APP_Swe_ KM670/671NL and human tau P301L. The steady-state levels of APP and tau proteins are nearly 3-4-fold and 6-8-fold higher in these mice as compared to endogenous levels in wild-type (WT) mice (Oddo et al., 2003a,b). These mice develop amyloid plaques and neurofibrillary tangle-like pathologies in a progressive and age-dependent manner, starting at ~9 and ~12 months, respectively, but show cognitive impairment as early as 2–3 months (Oddo et al., 2003b; Billings et al., 2005; Davis et al., 2013; Stevens and Brown, 2015). Several other aspects of the pathology in these mice also mimic AD pathophysiological changes and clinical phenotypes such as neurogenesis and synaptic plasticity impairments, and cognitive decline, all of which precede Aβ and tau pathologies (Oddo et al., 2003b; Billings et al., 2005).

The homozygous 3xTg-AD mice were obtained from Dr. Frank LaFerla (University of California, Irvine, CA, USA) through Jackson Laboratory (New Harbor, ME, USA). The background of the 3xTg-AD mice is a hybrid 129/Sv × C57BL/6. The non-transgenic WT mice used were from the same strain and genetic background and were also obtained from Jackson Laboratory. Mice were group-housed (4 animals per cage) with a 12:12 h light/dark cycle and with *ad libitum* access to food and water. This study was performed on 3-week-old homozygous 3xTg-AD (*n* = 147) and WT (*n* = 42) male and female mice. The mice were housed and bred as per the PHS Policy on Human Care and Use of Laboratory animals. The study protocols were approved by the Institutional Animal Care and Use Committee (IACUC) at SUNY Downstate Medical Center (Protocol ID: 13-10391) and the IACUC at New York State Institute for Basic Research (Protocol Number: ASP199).

### * In Vivo* Auditory Stimulation

Epileptogenic susceptibility to auditory stimuli was tested as described before (Zhong et al., 2009, 2010). Briefly, 20–22 day-old 3xTg-AD and WT mice were subjected to auditory stimulation for 5 min in a plastic cage with a high-pitched siren (120 dB) from a personal alarm device (TBO-Tech, Bonita Springs, FL, USA) mounted under a Styrofoam cage cover. Videos were recorded with a digital camcorder, and were analyzed by an experimenter blind to the animal genotype or treatment. Recorded parameters included the percentage of mice undergoing seizures (including tonic and clonic components, and status epilepticus) and the time to onset of seizure (latency). Seizure onset was defined as the moment when a mouse collapsed in convulsion.

To evaluate the effect of passive immunization against human APP/Aβ, the 14–15 day-old 3xTg-AD mice were injected via a single intraperitoneal (i.p.) injection with an anti-human APP/Aβ antibody, 6E10 (Covance, Princeton, NJ, USA), and audiogenic seizure susceptibility was tested when the mice were 21-day-old. The 6E10, a mouse monoclonal IgG-based antibody, was generated against amino acid residues 1–16 of human APP; the epitope lies with amino acids 3–8 of human APP. The antibody is specific to human APP/Aβ, and recognizes both precursor forms as well as abnormally processed isoforms of human APP, i.e., APP, sAPPα, and Aβ. Each litter of animals was divided into two groups. Animals in one group were injected i.p. with 40 μL of 0.9% NaCl (Saline), and the second group was injected with 6E10 (18.4 μg) in saline (40 μL). The dose, route of administration, and timing of passive immunization against human APP/Aβ were selected based on a previous study (Westmark et al., 2010).

For experiments with mGluR5 selective antagonist 2-methyl-6-(phenylethynyl)pyridine hydrochloride (MPEP; Tocris Bioscience, Minneapolis, MN, USA), each litter of ~3-week-old 3xTg-AD mice was divided into three groups. Mice in the first group were injected i.p. with 40 μL of saline; the second and third groups were injected with 40 μL of saline containing MPEP at concentrations 25 mg/Kg and 40 mg/Kg, respectively. MPEP solutions were freshly prepared before the injections. Mice were subjected to audiogenic stimulation 30 min after administration of MPEP or saline.

### Hippocampal Slice Preparations

The 3-week-old WT and 3xTg-AD mice were deeply anesthetized with isoflurane and euthanized via decapitation. The whole right cerebral hemisphere was immersion-fixed in 4% paraformaldehyde in 0.1 M phosphate buffered saline (PBS) for immunohistochemical studies. The hippocampus was dissected out from the left cerebral hemisphere, and transverse slices (400-μm-thick) were prepared from the isolated hippocampus using a vibratome (Lancer Series 1000, The Vibratome Company, Evergreen, St. Louis, MO, USA), as described before (Lee et al., 2002; Chuang et al., 2005; Zhong et al., 2009; Zhao et al., 2011; Osterweil et al., 2013). During slicing, the hippocampus was submerged in an ice-cold low-Ca^2+^/high-Mg^2+^ buffer containing the following (in mM): 124 NaCl, 26 NaHCO_3_, 2.5 KCl, 8 MgCl_2_, 0.5 CaCl_2_ and 10 D-glucose, continuously bubbled with 95% O_2_ and 5% CO_2_. For electrophysiology, slices were transferred to an interface recording chamber (Fine Science Tools, Vancouver, BC, Canada) perfused with artificial CSF (ACSF) consisting of the following (in mM): 124 NaCl, 26 NaHCO_3_, 5 KCl, 1.6 MgCl_2_, 2 CaCl_2_ and 10 D-glucose, bubbled with 95% O_2_ and 5% CO_2_; pH 7.4. Slices were maintained at 34–36°C for at least 60 min before recording.

### Electrophysiological Recordings

The electrophysiological techniques were used which have been described before (Chuang et al., 2005; Zhong et al., 2009; Zhao et al., 2011). Briefly, current clamp, intracellular recordings were performed in CA3 pyramidal cells (CA3a and CA3b sub-regions) using an Axoclamp 2A amplifier (Molecular Devices, Palo Alto, CA, USA). Microelectrodes were pulled from thin-walled glass tubes (1 mm outer diameter with glass filament inside; World Precision Instruments, Sarasota, FL, USA) using a micropipette puller (Sutter Instruments, Novato, CA, USA). Microelectrodes were filled with 2 M potassium acetate (typical resistances, 30–50 MΩ). An oscilloscope (DSO 400; Gould Instrument Systems, Cleveland, OH, USA) and a chart recorder (TA240; Gould Instrument Systems, Cleveland, OH, USA) were used for immediate display of voltage signals. These signals were digitized and stored in an Intel Pentium-based computer using Digidata 1322A converter controlled by pClamp 8 software (Molecular Devices, Palo Alto, CA, USA). CA3 pyramidal cells studied had stable resting membrane potentials of ~−60 mV and overshooting action potentials. In some instances, hyperpolarizing DC was injected into the cells to prevent intrinsic firing and identify network activities as spontaneous rhythmic depolarizations whose frequency was not affected by membrane potential changes.

### Pharmacological Treatments

Baseline epileptiform activities for WT and 3xTg-AD mouse hippocampal slices were elicited by continuous bath perfusion of the GABA_A_ receptor antagonist, bicuculline (50 μM; Sigma-Aldrich, St. Louis, MO, USA). The effect of the mGluR5 antagonist, MPEP (50 μM) on bicuculline-induced prolonged epileptiform (ictal-like) discharges in 3xTg-AD mice hippocampal slices was evaluated. For the passive immunization with 6E10 studies, 14–15 day-old 3xTg-AD mice were injected i.p. with saline or antibody in saline as described above for audiogenic seizure susceptibility studies, and hippocampal slices experiments were performed on 21-day-old mice.

### Tissue Processing

The whole right cerebral hemisphere from WT and 3xTg-AD mice from electrophysiology studies was immediately immersion fixed in 4% paraformaldehyde in 0.1 M PBS for 24–48 h, followed by cryoprotection in a 30% sucrose solution at 4°C overnight. Later, 40 μm-thick sagittal sections were cut on a freezing microtome. The sections were stored in glycol anti-freeze solution (ethylene glycol, glycerol, and 0.1 M PBS in a 3:3:4 ratio) at −20°C till further processing for immunohistochemical staining.

### Immunohistochemistry

Immunohistochemical studies for evaluation of human APP/Aβ expression were performed on free-floating brain sections, as described previously (Kazim et al., 2014; Chohan et al., 2015; Dai et al., 2015). Every 8–9th brain section was chosen for densitometric quantification which was carried out using a minimum of five brain sections/mouse from five to six animals/group. All stainings for fluorescence intensity quantification were carried out under similar conditions including all tissue sections for an experiment stained at the same time, and imaged using identical laser power and detector settings. The following primary antibodies at the indicated dilutions were employed: mouse monoclonal anti-human APP/Aβ antibody, 6E10 [1:200; recognizes N-terminal amino acids 1–16 of Aβ peptide (epitope lies within amino acids 3–8 of Aβ), sAPPα, and C-terminal fragment (CTF)β/C99, and stains human APP and Aβ; Covance, Princeton, NJ, USA], and rabbit polyclonal anti-human APP antibody, CT20 [1:500; C-terminal (751–770), recognizes full-length APP, and CTFα, CTFβ, and CTFγ, and stains human APP/CTFs; EMD Millipore, Billerica, MA, USA]. The following secondary antibodies were used: Alexa 488-conjugated goat anti-mouse IgG anti-body (1:500, Molecular Probes, Carlsbad, CA, USA), and Alexa 488-conjugated goat anti-rabbit IgG antibody (1:500, Molecular Probes, Carlsbad, CA, USA). Maximum projection images of the CA3 region of the hippocampus in stained sections were generated based on confocal *z*-stacks using Nikon 90i fluorescent microscope equipped with Nikon C1 three-laser confocal system and a Nikon DS U1 digital camera. For densitometry, the whole CA3 region was outlined, and quantified by measuring mean pixel intensity (MPI) with the software Image Pro Plus 5.0 (Media Cybernetics, Silver Spring, MD, USA), as described previously (Kazim et al., 2014).

### Data Analysis

For audiogenic seizure susceptibility experiments, the incidence of seizures data is presented as percent incidence with 95% confidence interval, and was analyzed using exact logistic regression analysis stratified by litter. The latency to seizure data is depicted as median with range (skewed distribution). Mann-Whitney *U* test, or for multiple groups, Kruskal-Wallis test followed by Dunn’s multiple comparison test, was employed for analyzing latency data. The D’Agostino-Pearson, Shapiro-Wilk, and Kolmogorov-Smirnov normality tests were used to determine if the data was normally distributed or skewed. The statistical analysis was performed using SAS v.9.4 (SAS Institute, Cary, NC, USA) and GraphPad Prism v 7.0 (GraphPad Software Inc., La Jolla, CA, USA). For all purposes, *p* < 0.05 was considered as statistically significant.

For electrophysiology data, the durations of individual synchronized discharges were measured from the beginning of the first action potential to the repolarization of the last action potential of the discharge. Membrane potentials were kept within a few millivolts throughout the experiment. Frequency histograms included the durations of all synchronized discharges that were recorded in 5 min periods for each slice under respective experimental conditions. Based on the frequency distribution of the synchronized discharges durations observed in previous studies (Chuang et al., 2005; Zhong et al., 2009), “short” burst discharges refer to synchronized discharges shorter than or equal to 1.5 s and “prolonged” epileptiform (ictal-like) discharges to those longer than 1.5 s. In the frequency histogram plots, the durations of all synchronized epileptiform discharges recorded in a 5-min period for each slice in the various experimental conditions were included. The histograms were fitted with first- or second-order Gaussian equations, as appropriate. The data on the average durations of the five longest synchronized discharges at respective time intervals were analyzed using repeated measures, two-way analysis of variance (ANOVA) followed by Bonferroni’s *post hoc* test. The comparisons involving average duration of the five longest discharges between study groups were performed using Student’s *t*-test. For passive immunization with 6E10 experiments, the data on percentage of slices with prolonged epileptiform discharges was analyzed using Fischer’s exact test and chi-square test with Yates correction. Also, for passive immunization with 6E10 experiments, latency to the occurrence of prolonged epileptiform discharges was analyzed using Student’s *t*-test. Clampfit v.9.2 (Axon Instruments/Molecular Devices, Novato, CA, USA), SPSS v.17.0 (SPSS Inc., Chicago, IL, USA) and GraphPad Prism 7.0 (GraphPad Software Inc., LaJolla, CA, USA) were used for data and statistical analysis. For all comparisons, *p* < 0.05 was set as statistical significance level.

For immunohistochemistry data, MPI was compared using Student’s *t*-test. For correlation data, Pearson correlation coefficient was calculated, and curve fitting was carried out by employing non-linear regression model. GraphPad Prism 7.0 (GraphPad Software Inc., LaJolla, CA, USA) was used for statistical analysis, with *p* < 0.05 considered as statistical significance level.

## Results

### Young 3xTg-AD Mice Are Highly Susceptible to Audiogenic Seizures

**Figure 1:**
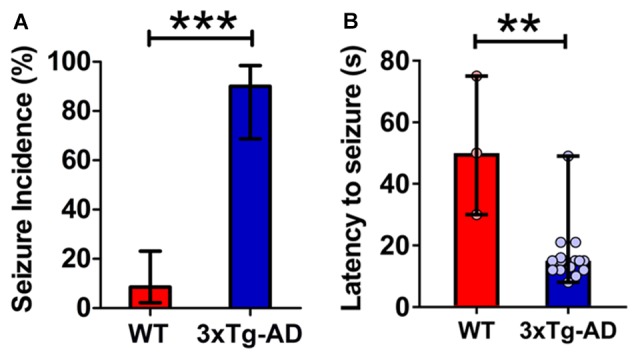
**Increased propensity for audiogenic seizures in 3-week-old triple transgenic mouse model of familial Alzheimer’s disease (3xTg-AD) mice. (A)** The incidence of convulsive seizures was markedly higher in 3xTg-AD mice (blue bar) compared to wild type (WT) mice (red bar). The data are presented as percent incidence with 95% confidence interval and compared using exact logistic regression stratified by litter. **(B)** The latency to seizure was significantly lower in 3xTg-AD mice as compared to WT control. The data are depicted as median with range, and analyzed using Mann-Whitney *U* test. **p < 0.01, ***p < 0.001, compared to WT. WT (*n* = 35) and 3xTg-AD (*n* = 20) mice.

In transgenic models of AD, network hypersynchrony and epileptic susceptibility have been shown to appear prior to amyloid plaque deposition, neurofibrillary pathology and cognitive dysfunction (Bezzina et al., 2015; Duffy et al., 2015; Kam et al., 2016; Shah et al., 2016). To probe for *in vivo* epileptogenic susceptibility prior to extensive neuropathology and cognitive deficit in 3xTg-AD mice, we exposed 3-week-old mice to 120 dB siren in a single 5-min-session and analyzed the percentage of animals exhibiting seizure and the latency to the occurrence of seizure (Figure 1; WT, *n* = 35; 3xTg-AD, *n* = 20; age = 20–22 days).

3xTg-AD mice typically exhibited early indications of epileptic activity in the form of wild, uncontrolled running and jumping. This frenzied, excessive motor activity was the first sign of imminent convulsive seizure. A remarkably higher percentage of 3xTg-AD mice (90%, 18 out of 20) developed tonic-clonic convulsive seizures as compared to WT controls (8.6%, 3 out of 35) within 1 min of the onset of alarm (Figure 1A; *p* < 0.001, exact logistic regression stratified by litter). In mice that developed convulsive seizures, the latency to the occurrence of seizures was markedly lower in 3xTg-AD mice as compared to WT mice (Figure 1B; *p* = 0.0038; Mann-Whitney *U* test).

These data show that: (1) young 3xTg-AD mice harboring mutated APP, tau and PS1 genes are acutely susceptible to audiogenic seizures; and (2) epileptogenic vulnerability appears before overt cognitive impairment and much before the development of extensive AD neuropathology in these mice.

### Young 3xTg-AD Mice Exhibit Hyperexcitability of the Hippocampal CA3 Neuronal Network

**Figure 2:**
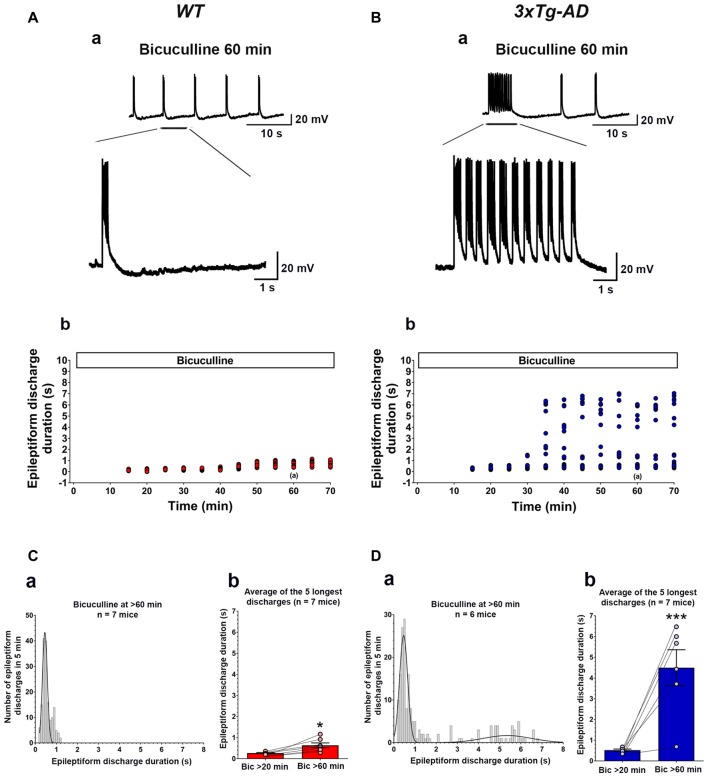
**Ictal-like epileptiform discharges in CA3 pyramidal cells of hippocampal slices from 3-week-old 3xTg-AD mice. (A)** CA3 intracellular recording from a WT slice after bicuculline addition (50 μM). Within 20 min, bicuculline induced rhythmic, short epileptiform discharges (≤1.5 s in duration) that were ongoing for at least 1 h of continuous recording. A representative trace of short epileptiform discharges from a WT slice at 60 min after bicuculline application is shown in **(Aa)**. Membrane potential at the beginning of recording: −60 mV. **(Ab)** Plot of the duration of discharges recorded in the cell shown in **(Aa)**. **(B)** CA3 intracellular recording from a 3xTg-AD slice after bicuculline. Bicuculline first induced short synchronized epileptiform discharges that were like those in WT slices. However, continuous perfusion with bicuculline induced prolonged epileptiform (ictal-like) discharges (>1.5 s) in 3xTg-AD slice **(Ba)**. Membrane potential at the beginning of recording: −65 mV.** (Bb)** Plot of the duration of discharges recorded in the cell shown in **(Ba)**. **(Ca)** Frequency histogram of the duration of all epileptiform discharges during a 5-min period after 60 min of bicuculline perfusion in WT mice (*n* = 7 mice, *n* = 11 slices). In WT slices, epileptiform discharges were normally distributed with an average duration of 0.560 ± 0.017 s (first-order Gaussian fit; *r* = 0.81). No prolonged epileptiform discharge was observed in WT slices. **(Cb)** The average duration of the five longest synchronized epileptiform discharges recorded during 5-min intervals at 20 min (0.267 ± 0.021 s) and 60 min (0.636 ± 0.114 s) after bicuculline application in WT slices. The short epileptiform discharges increased in duration significantly over the time course of the recordings in WT slices, however, no prolonged discharge (>1.5 s) was observed. **(Da)** Frequency histogram of the duration of all synchronized epileptiform discharges during a 5-min-period after 60 min of bicuculline perfusion in 3xTg-AD mice (*n* = 6 mice, *n* = 9 slices). A two-peak distribution showed a group of short epileptiform discharges with average duration of 0.572 ± 0.024 s and a group of prolonged epileptiform discharges of 4.664 ± 0.204 s (second-order Gaussian fit; *r* = 0.79). **(Db)** The average duration of the five longest synchronized discharges recorded during 5 min intervals after 60 min (4.497 ± 0.869) was significantly higher than that after 20 min (0.521 ± 0.051) of bicuculline perfusion. *p < 0.05, ***p < 0.001, repeated measures two-way analysis of variance (ANOVA).

The earliest neuropathological changes in AD occur in the hippocampus and entorhinal cortex, followed by changes in the medial temporal lobe (Belleville et al., 2008; Reitz et al., 2009; Small et al., 2011). Correspondingly, the earliest known cognitive deficit in AD is in the hippocampus-dependent episodic memory including its crucial features, pattern separation and pattern completion (Collie and Maruff, 2000; Ally et al., 2013). The CA3 region of the hippocampus is known to play a major role in both pattern separation and pattern completion (Yassa and Stark, 2011). We thus evaluated the CA3 pyramidal neuronal excitability and epileptogenic susceptibility using pharmacological disinhibition of hippocampal slices as an *in vitro* model of epileptogenesis (Chuang et al., 2005). Intracellular recordings were carried out in CA3 pyramidal neurons of hippocampal slice preparations isolated from 3-week-old 3xTg-AD mice and their WT controls (Figure 2). Spontaneous epileptiform discharges were elicited by application of the GABA_A_ receptor antagonist, bicuculline. Previous studies have shown that in slices from WT animals, this treatment typically induces short (≤1.5 s in duration), interictal-like epileptiform discharges (Prince and Wong, 1981; Chuang et al., 2005), whereas in slices from transgenic mice that exhibit enhanced seizure susceptibility bicuculline can induce prolonged (>1.5 s), ictal-like epileptiform discharges (Chuang et al., 2005; Zhong et al., 2009, 2010).

Bath application of bicuculline (50 μM) to hippocampal slice preparations prepared from WT mice (*n* = 11 slices from *n* = 7 mice) caused the appearance, within 20 min, of regular and rhythmic short synchronized discharges (mean duration = 0.215 ± 0.004 s) in the CA3 area (Figures 2A,C). The duration of short synchronized discharges remained ≤ 1.5 s for at least 1 h in all WT slice preparations studied (Figures 2A,C). The average duration of short synchronized discharges recorded 60 min after bicuculline application was 0.560 ± 0.017 s (Figure 2Ca; first-order Gaussian fit; *r* = 0.81). The average durations of the five longest synchronized discharges during a 5-min-period recorded 60 min after bicuculline application in WT slices were significantly prolonged as compared to those recorded after 20 min (Figure 2Cb; *p* = 0.016, repeated measures, two-way ANOVA). However, no prolonged ictal-like epileptiform discharge (>1.5 s) was observed during the period of recording in WT slices.

Figures 2B,D depicts data obtained from hippocampal slices prepared from 3xTg-AD mice (*n* = 9 slices from *n* = 6 mice). Like in WT slices, bicuculline (50 μM) application induced rhythmic, short interictal-like epileptiform discharges. The average duration of these short epileptiform synchronized discharges 20 min after bicuculline application was 0.395 ± 0.008 s. Hippocampal slices from the majority of 3xTg-AD mice (5/6, 83.3%) started to show a distinct population of prolonged ictal-like epileptiform discharges (>1.5 s) about 30 min after bicuculline application (Figures 2B,D). The average duration of these prolonged epileptiform discharges was 4.664 ± 0.204 s as compared to the mean duration of 0.576 ± 0.024 s of the population of short epileptiform discharges (Figure 2Da; second-order Gaussian fit; *r* = 0.79). The average durations of the five longest synchronized epileptiform discharges recorded during 5-min periods after 60 min of bicuculline perfusion were significantly prolonged compared to those recorded after 20 min of bicuculline (Figure 2Db; *p* < 0.001, repeated measures, two-way ANOVA). Also, the average duration of five longest epileptiform discharges during 5-min periods after 60 min of bicuculline was significantly higher in slices from 3xTg-AD mice as compared to WT controls (*p* = 0.0006; Student’s *t*-test). These data indicate that at 3 weeks of age, 3xTg-AD mice exhibit marked hippocampal CA3 neuronal hypersynchronous, ictal-like activity.

### Expression of Intraneuronal Human APP/Aβ Is Seen in the Hippocampal CA3 Region of Young 3xTg-AD Mice

**Figure 3:**
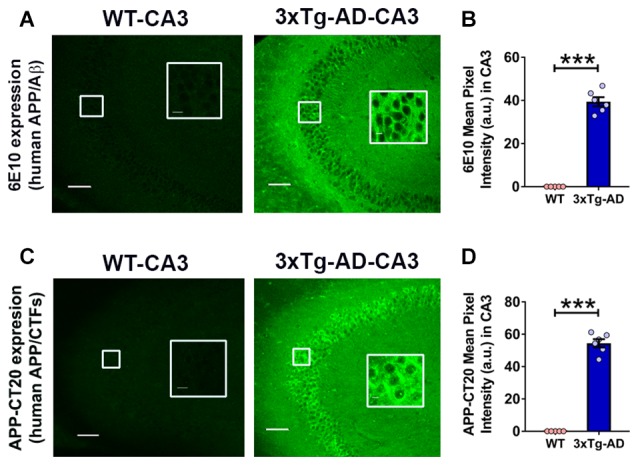
**Expression of intraneuronal human amyloid β precursor protein (APP)/amyloid beta (Aβ) in the hippocampal CA3 region of 3-week-old 3xTg-AD mice. (A)** Representative photomicrographs showing 6E10 (human APP/Aβ) intraneuronal expression in the hippocampal CA3 region of 3-week-old 3xTg-AD mice; as expected WT mice did not display 6E10 immunoreactivity. No extracellular amyloid plaque deposition was observed. **(B)** Densitometric quantification of the immunohistochemistry data with 6E10 expression. **(C)** Representative photomicrographs showing APP-CT20 (human APP/C-terminal fragments, CTFs) intraneuronal expression in the hippocampal CA3 region of 3-week-old 3xTg-AD mice; no expression is observed in WT mice. **(D)** Densitometric quantification of the immunohistochemistry data with APP-CT20 expression. The data are shown as mean ± SEM from WT (*n* = 5) and 3xTg-AD (*n* = 6), and compared using Student’s *t*-test. Insets show the designated areas at higher magnification. Scale bar = 100 μm. ***p < 0.001.

The 3xTg-AD mice carry a 3–4 fold APP overexpression (Oddo et al., 2003a). The intraneuronal human APP/Aβ immunoreactivity in the CA fields of the hippocampus has been documented in 3xTg-AD mice as early as 3 weeks of age (Billings et al., 2005; Oh et al., 2010). We evaluated the human APP/Aβ expression in the CA3 region of the hippocampus in 3-week-old 3xTg-AD mice and WT controls by employing two different antibodies (Figure 3). First, we used anti-APP/Aβ antibody, 6E10, which recognizes N-terminal amino acids 1–16 of the Aβ peptide and stains human APP and Aβ. The 6E10 antibody also recognizes sAPPα and CTFβ, C99 (Chang et al., 2003). The 6E10 staining revealed immunoreactive neurons in the CA3 region of 3-week-old 3xTg-AD mice, and as expected, the WT mice failed to show immunoreactivity (Figures 3A,B; *p* < 0.001, Student’s *t*-test). Second, we employed anti-human APP antibody, APP-CT20, which recognizes full-length APP and CTFs. Like 6E10, the APP-CT20 staining showed immunoreactivity in the CA3 region of 3-week-old 3xTg-AD mice, and not in WT mice (Figures 3C,D; *p* < 0.001, Student’s *t*-test).

Taken together, these data confirmed the intraneuronal human APP/Aβ immunoreactivity as early as 3 weeks of age in 3xTg-AD mice. As expected, no extracellular amyloid plaque deposits were found at this age in 3xTg-AD mice.

### Passive Immunization with the Anti-Human APP/Aβ Antibody, 6E10, Attenuates Seizure Susceptibility in Young 3xTg-AD Mice

**Figure 4:**
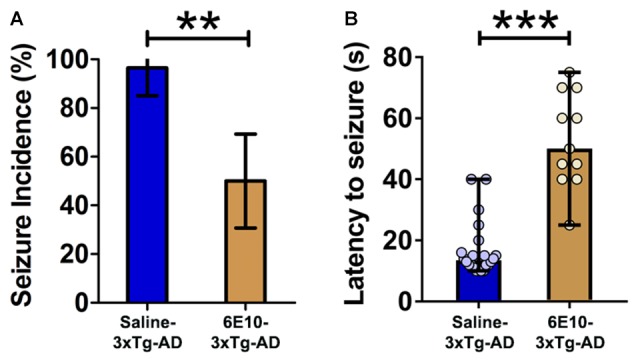
**Attenuation of audiogenic seizure susceptibility by passive immunization with anti-human APP/Aβ antibody, 6E10, in 3-week-old 3xTg-AD mice. (A)** The incidence of convulsive seizures in 3xTg-AD mice was significantly reduced by passive immunization with 6E10. The data are presented as percent incidence with 95% confidence interval and compared using exact logistic regression stratified by litter. **(B)** Passive immunization with 6E10 also significantly increased the latency to seizure in 3xTg-AD mice. The data are depicted as median with range, and analyzed using Mann-Whitney *U* test. **p < 0.01, ***p < 0.001, compared to Saline-3xTg-AD. Saline-3xTg-AD (*n* = 23) and 6E10–3xTg-AD (*n* = 22) mice.

Genetic suppression of transgenic human APP was shown to suppress hypersynchronous network activity in an AD mouse model (Born et al., 2014). Also, intraneuronal Aβ accumulation was reported to induce hippocampal neuron hyperexcitability (Scala et al., 2015). We thus sought to determine if pharmacological clearance of intraneuronal human APP/Aβ could ameliorate the seizure susceptibility in young 3xTg-AD mice. We assessed audiogenic seizure rate after passive immunization with an anti-human APP/Aβ monoclonal antibody, 6E10, in 3-week-old 3xTg-AD mice (Figure 4; Saline-3xTg-AD, *n* = 23, 6E10–3xTg-AD, *n* = 22).

Passive immunization with 6E10 significantly reduced the seizure incidence in 3-week-old 3xTg-AD mice (Figure 4A; *p* < 0.01; exact logistic regression stratified by litter). Passive immunization with 6E10 also increased significantly the latency to convulsive seizures (Figure 4B; *p* < 0.001, Mann-Whitney *U* test). Overall, these data suggest that passive immunization against human APP/Aβ decreases the susceptibility to audiogenic seizures in young 3xTg-AD mice.

### Passive Immunization with Anti-Human APP/Aβ Antibody, 6E10, Decreases Intraneuronal Human APP/Aβ Expression in Young 3xTg-AD Mice

**Figure 5:**
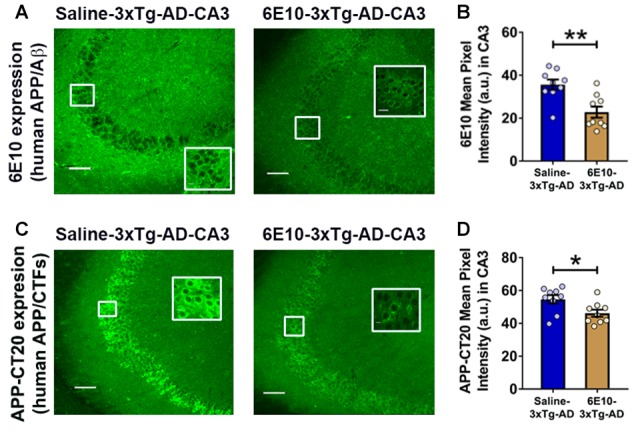
**Reduction in intraneuronal human APP/Aβ expression by passive immunization with anti-human APP/Aβ antibody, 6E10, in the hippocampal CA3 region of 3-week-old 3xTg-AD mice. (A)** Representative photomicrographs illustrating reduction in 6E10 (human APP/Aβ) intraneuronal immunoreactivity in the hippocampal CA3 region of young 3xTg-AD mice after passive immunization with 6E10. **(B)** Densitometric quantification of the immunohistochemistry data with 6E10 expression. **(C)** Representative photomicrographs showing decrease in APP-CT20 (human APP/CTFs) intraneuronal expression in the hippocampal CA3 region of young 3xTg-AD mice after passive immunization with 6E10. **(D)** Densitometric quantification of the immunohistochemistry data with APP-CT20 expression. The data are shown as mean ± SEM from Saline-3xTg-AD (*n* = 9) and 6E10–3xTg-AD (*n* = 9), and compared using Student’s *t*-test. Insets show the designated areas at higher magnification. Scale bar = 100 μm. *p < 0.05, **p < 0.01.

Previously, treatment with 6E10 was demonstrated to reduce intraneuronal human Aβ and cell-surface full-length APP in cultured neurons from Tg2576 mice carrying hAPP_SWE_ mutation (Tampellini et al., 2007). We evaluated the effect of passive immunization with 6E10 on human APP/Aβ expression in the hippocampal CA3 neurons of 3-week-old 3xTg-AD mice (Figure 5). Densitometric quantification revealed that passive immunization with 6E10 significantly reduced human APP/Aβ expression, as analyzed by 6E10 expression, in the CA3 region of young 3xTg-AD mice (Figures 5A,B; *p* = 0.0023, Student’s *t*-test). Furthermore, APP-CT20 staining also revealed a significant reduction in human APP/CTFs expression (Figures 5C,D; *p* = 0.0228, Student’s *t*-test). These data suggest that 6E10 passive immunization-induced reduction in seizure susceptibility in young 3xTg-AD mice may result from the reduction in transgenic human APP/Aβ.

### Passive Immunization with Anti-Human APP/Aβ Antibody, 6E10, Reduces Ictal-Like Activity in Hippocampal Slices from Young 3xTg-AD Mice

**Figure 6:**
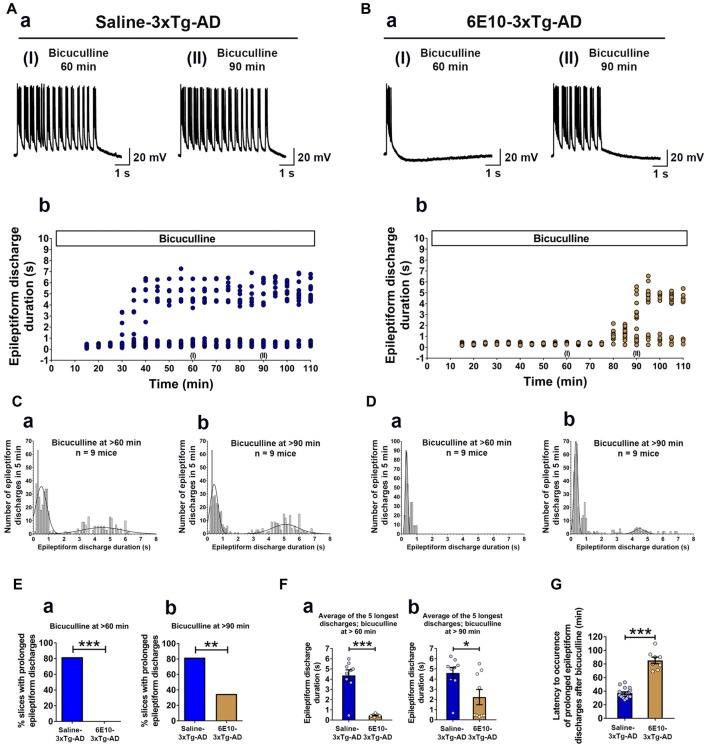
**Suppression of ictal-like activity by passive immunization with anti-human APP/Aβ antibody, 6E10, in hippocampal slices from 3-week-old 3xTg-AD mice. (A)** CA3 intracellular recordings from a Saline-3xTg-AD slice after bicuculline application (50 μM). Continuous perfusion with bicuculline induced prolonged synchronized epileptiform discharges (>1.5 s) in Saline-3xTg-AD slice which are depicted at 60 min **(AaI)** and 90 min **(AaII)** after bicuculline application. Membrane potential at the beginning of recordings: −73 mV (60 min) and −75 mV (90 min).** (Ab)** Plot of the duration of epileptiform discharges recorded in the cell shown in **(Aa)**. **(B)** CA3 intracellular recordings from a 6E10–3xTg-AD slice revealed only short epileptiform discharges 60 min after bicuculline application **(BaI)**. Fully developed prolonged epileptiform discharges were not observed until 90 min after bicuculline **(BaII)**. Membrane potential at the beginning of recordings: −76 mV (60 min) and −74 mV (90 min). **(Bb)** Plot of the duration of epileptiform discharges recorded in the cell shown in **(Ba)**. **(C)** Frequency histograms of the duration of all synchronized discharges during a 5-min period after 60 min **(Ca)** and 90 min **(Cb)** of bicuculline perfusion in Saline-3xTg-AD mice (*n* = 22 slices from *n* = 9 mice). At 60 min after bicuculline, a two-peak distribution showed a group of short epileptiform discharges with average duration of 0.560 ± 0.015 s and a group of prolonged epileptiform discharges of 4.339 ± 0.086 s (second-order Gaussian fit; *r* = 0.79). Also, at 90 min after bicuculline, a two-peak distribution showed a group of short epileptiform discharges with average duration of 0.553 ± 0.018 s and a group of prolonged epileptiform discharges of 4.951 ± 0.079 s (second-order Gaussian fit; *r* = 0.83). **(D)** Frequency histograms of the duration of all epileptiform discharges during a 5-min period after 60 min **(Da)** and 90 min **(Db)** of bicuculline perfusion in 6E10–3xTg-AD mice (*n* = 23 slices from *n* = 9 mice). At the 60-min time point, all epileptiform discharges were normally distributed with an average duration of 0.475 ± 0.011 s (first-order Gaussian fit; *r* = 0.91). No prolonged epileptiform discharge was observed in Saline-3xTg-AD mice at 60 min after bicuculline application. However, some of the slices (8 out of 23, 34.8%) exhibited long epileptiform discharges 90 min after bicuculline. At 90 min after bicuculline, a two-peak distribution showed a group of short epileptiform discharges with average duration of 0.466 ± 0.016 s and a group of prolonged epileptiform discharges of 4.195 ± 0.168 s (second-order Gaussian fit; *r* = 0.88). **(E)** The overall incidence of prolonged epileptiform discharges both 60 min **(Ea)** and 90 min **(Eb)** after bicuculline application was significantly reduced by passive immunization with 6E10 (*p* < 0.001 and *p* = 0.0023, respectively; Fischer’s exact test). **(F)** Passive immunization with 6E10 significantly reduced the average durations of the five longest synchronized epileptiform discharges recorded during 5 min intervals both 60 min **(Fa)** and 90 min **(Fb)** after bicuculline (*p* < 0.001 and *p* = 0.021, respectively; Student’s *t*-test). **(G)** The latency to occurrence of prolonged epileptiform discharges was significantly higher in 6E10–3xTg-AD mice (85.3 ± 4.8 min) as compared to Saline-3xTg-AD mice (37.2 ± 1.7 min; *p* < 0.001; Student’s *t*-test). *p < 0.05, **p < 0.01, ***p < 0.001.

We further evaluated the effect of passive immunization with anti-human APP/Aβ antibody, 6E10, on ictal-like activity in the CA3 pyramidal neurons of the hippocampal slices from 3-week-old 3xTg-AD mice (Figure 6). CA3 intracellular recordings revealed that bath application of bicuculline (50 μM) to hippocampal slice preparations from Saline-3xTg-AD mice (*n* = 22 slices from *n* = 9 mice) induced regular and rhythmic short epileptiform discharges (mean duration = 0.345 ± 0.006 s) within 20 min (Figures 6A,C). Prolonged epileptiform discharges (>1.5 s) started appearing at >30 min after bicuculline application in the majority of slices from Saline-3xTg-AD mice (Figures 6A,C). At 60 min after bicuculline, a two-peak distribution showed a group of short epileptiform discharges with average duration of 0.560 ± 0.015 s and a group of prolonged epileptiform discharges of 4.339 ± 0.086 s (second-order Gaussian fit; *r* = 0.79; Figure 6Ca). Also, at 90 min after bicuculline, a two-peak distribution showed a group of short epileptiform discharges with average duration of 0.553 ± 0.018 s and a group of prolonged epileptiform discharges of 4.951 ± 0.079 s (second-order Gaussian fit; *r* = 0.83; Figure 6Cb). The average durations of the five longest synchronized discharges from Saline-3xTg-AD mice recorded during 5-min periods after 60 min (4.369 ± 0.540 s; Figure 6Fa, *blue bar*) and 90 min (4.594 ± 0.552 s; Figure 6Fb, *blue bar*) were significantly longer than those after 20 min (0.301 ± 0.025 s) of bicuculline perfusion (*p* < 0.001, repeated measures, two-way ANOVA).

Remarkably, in contrast to slices from saline injected 3xTg-AD mice, none of the slices from 6E10–3xTg-AD mice (*n* = 23 slices from *n* = 9 mice) exhibited prolonged epileptiform discharges 60 min after bicuculline application (Figures 6B,D,Ea; mean duration = 0.475 ± 0.010 s, first-order Gaussian fit; *r* = 0.91). However, 90 min after bicuculline application, 34.8% of the slices from 6E10–3xTg-AD mice developed prolonged epileptiform discharges (Figures 6B,D,Eb). At 90 min after bicuculline, a two-peak distribution showed a group of short epileptiform discharges with average duration of 0.466 ± 0.016 s and a group of prolonged epileptiform discharges of 4.195 ± 0.168 s (second-order Gaussian fit; *r* = 0.88; Figure 6Db). The average duration of the five longest synchronized epileptiform discharges from 6E10–3xTg-AD mice recorded during a 5-min-period after 90 min (2.232 ± 0.747 s; Figure 6Fb, brown bar) was significantly longer than those after 20 min (0.297 ± 0.020 s) and 60 min (0.440 ± 0.041 s; Figure 6Fa, brown bar) of bicuculline perfusion (*p* < 0.01, repeated measures, two-way ANOVA).

The overall incidence of bicuculline-induced prolonged epileptiform discharges was significantly reduced by passive immunization with 6E10 (Figure 6Ea; 60 min after bicuculline: 18 out of 22 (81.8%) slices from Saline-3xTg-AD mice (*n* = 9) vs. 0 out of 23 (0%) slices from 6E10–3xTg-AD mice (*n* = 9), Fischer’s exact test, *p* < 0.001, Chi-square test with Yates correction, *p* < 0.001; Figure 6Eb; 90 min after bicuculline: 18 out of 22 (81.8%) slices from Saline-3xTg-AD mice (*n* = 9) vs. 8 out of 23 (34.8%) slices from 6E10–3xTg-AD mice (*n* = 9), Fischer’s exact test, *p* = 0.0023, Chi-square test with Yates correction, *p* = 0.0038).

Passive immunization with 6E10 significantly reduced the average of the five longest epileptiform discharges both at 60 and 90 min time points after bicuculline application (Figures 6Fa,b; bicuculline 60 min, Saline-3xTg-AD, 4.369 ± 0.545 s, vs. 6E10–3xTg-AD, 0.440 ± 0.041 s, *p* < 0.001, Student’s *t*-test; and bicuculline 90 min, Saline-3xTg-AD, 4.954 ± 0.552 s, vs. 6E10–3xTg-AD, 2.232 ± 0.747, *p* = 0.021; Student’s *t*-test). Remarkably, in the slices that developed prolonged epileptiform discharges, passive immunization with 6E10 significantly increased the latency to the occurrence of these discharges (Figure 6G; Saline-3xTg-AD mice, 37.3 ± 1.7 min, vs. 6E10–3xTg-AD mice, 85.2 ± 4.8 min; *p* < 0.001, Student’s *t*-test).

Overall, these data show that passive immunization with anti-human APP/Aβ antibody, 6E10, significantly reduced ictal-like activity in the CA3 neuronal network of the hippocampus. The delay in the appearance of prolonged epileptiform discharges in some of the 6E10-injected 3xTg-AD mice may suggest a role for intraneuronal human APP/Aβ in the induction of hypersynchronous network activity.

### The Ictal-Like Activity in CA3 Hippocampal Region Correlates Positively with Intraneuronal APP/Aβ Expression in Young 3xTg-AD Mice

**Figure 7:**
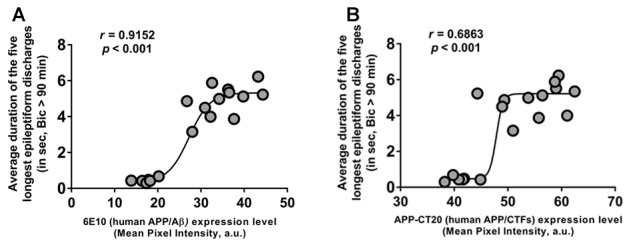
**Positive correlation of intraneuronal human APP/Aβ expression in CA3 neurons and ictal-like activity in CA3 region.** **(A,B)** Correlation analyses revealed a positive relationship between intraneuronal human APP/Aβ immunoreactivity in the CA3 neurons (analyzed by 6E10, human APP/Aβ **(A)** and by APP-CT20, human APP/CTFs **(B)**) and average duration of the five longest epileptiform discharges recorded during a 5-min period after 90 min of bicuculline application in CA3 region of hippocampal slices from the same mice. Data from Saline-3xTg-AD (*n* = 9) and 6E10–3xTg-AD (*n* = 9) was pooled together to evaluate the correlations depicted in **(A,B)**. The sigmoidal curves based on non-linear regression are also shown.

To further evaluate the role of human APP/Aβ in epileptiform activities in 3-week-old 3xTg-AD mice, we performed correlation analyses between the duration of the epileptiform discharges recorded from CA3 neurons and the human APP/Aβ expression level in the CA3 region in the brains of the same mice. The data from the Saline-3xTg-AD and 6E10–3xTg-AD mice were pooled together for the correlation analyses. We found a significant positive correlation between intraneuronal human APP/Aβ expression level (both 6E10 and APP-CT20) and ictal-like activity (Figures 7A,B; 6E10 expression and ictal-like activity, Pearson correlation coefficient, *r* = 0.9152, *p* < 0.001; APP-CT20 expression and ictal-like activity, Pearson correlation coefficient, *r* = 0.6893, *p* < 0.001). These data strongly support the role of human (transgenic) APP/Aβ in ictal-like activity in young 3xTg-AD mice.

### The mGluR5 Antagonist MPEP Suppresses Convulsive Audiogenic Seizures in Young 3xTg-AD Mice

**Figure 8:**
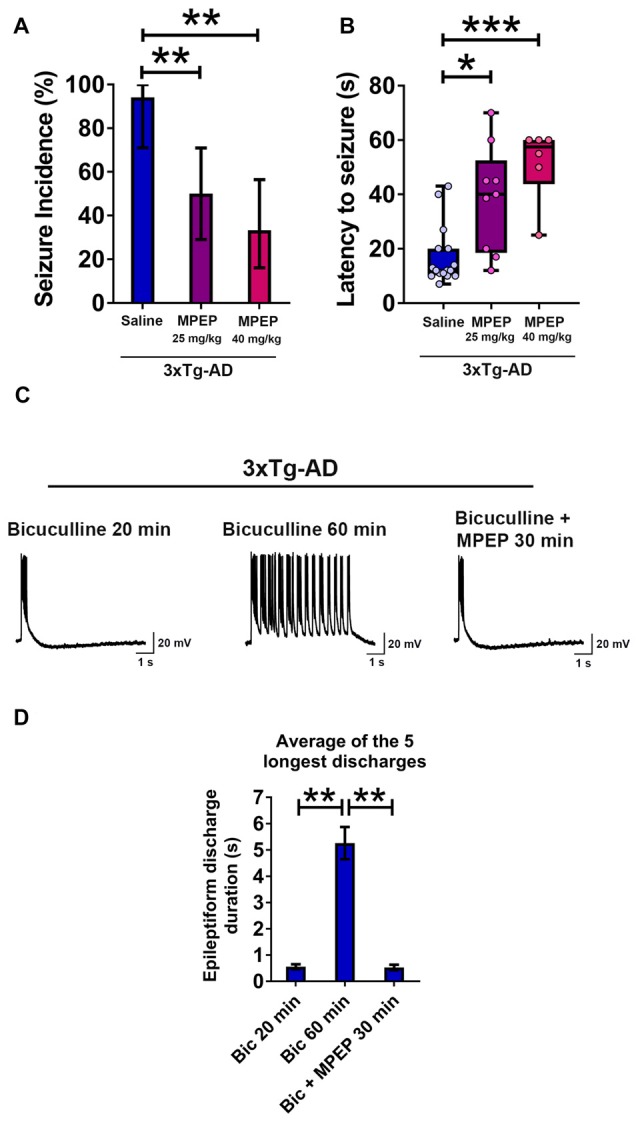
**Suppression of seizure susceptibility and prolonged epileptiform discharges in hippocampal slices of 3-week-old 3xTg-AD mice by the metabotropic glutamate receptor 5 (mGluR5) antagonist 2-methyl-6-(phenylethynyl)pyridine hydrochloride (MPEP). (A)** The incidence of convulsive seizures was significantly reduced in a dose dependent manner by intraperitoneal (i.p.) injection of MPEP at 25 mg/kg or 40 mg/kg, half an hour prior to audiogenic stimulation, compared to a saline injected group. The data are presented as percent incidence with 95% confidence interval and compared using exact logistic regression stratified by litter. **(B)** The data are depicted as box and whisker plots (median with interquartile range, and upper and lower extremes) and analyzed using Kruskal-Wallis test followed by Dunn’s multiple comparison test. The data shown in **(A,B)** are based on the following groups: saline control, *n* = 17; MPEP 25 mg/kg, *n* = 18; and MPEP 40 mg/kg, *n* = 18. **(C,D)** Prolonged epileptiform discharges in hippocampal CA3 pyramidal neurons of slices from 3-week-old 3xTg-AD mice recorded at 60 min after bicuculline perfusion were suppressed by MPEP (50 μM). Data are presented as mean ± SEM of the five longest epileptiform discharges from *n* = 9 slices from *n* = 5 mice, and compared using two-way ANOVA followed by Bonferroni *post hoc* test. *p < 0.05, **p < 0.01, ***p < 0.001.

Our above data indicate that intraneuronal human APP/Aβ is a likely cause of neuronal hyperexcitability and propensity to epileptic seizures in young 3xTg-AD mice. Activation of the mGluR5 has been shown to increase translation of APP mRNA (Westmark and Malter, 2007; Sokol et al., 2011; Westmark, 2013). The mGluR5 activation is also known to mediate Aβ oligomer-induced neuronal and synaptic dysfunction (Renner et al., 2010; Um et al., 2013). Blockade of mGuR5 with the noncompetitive antagonist MPEP which can cross the blood-brain barrier (Gasparini et al., 1999; Varney et al., 1999) has been shown to attenuate seizure susceptibility in mouse models of AD and DS (Westmark et al., 2010), and fragile X syndrome (FXS; Yan et al., 2005; Westmark et al., 2009, 2010; Zhong et al., 2009). We thus evaluated the effect of MPEP on audiogenic seizure susceptibility to determine whether mGluR5 activation plays a role in this ictal-like activity mediated behavioral phenotype in 3-week-old 3xTg-AD mice (Figures 8A,B).

We administered i.p. MPEP 30 min before auditory stimulation. MPEP-injected 3xTg-AD mice exhibited a decrease in the incidence of audiogenic seizures (Figure 8A; MPEP 25 mg/Kg, *p* < 0.01; MPEP 40 mg/Kg, *p* < 0.01; exact logistic regression stratified by litter). MPEP-injected 3xTg-AD mice exhibited a significant, dose-dependent increase in the latency to occurrence of convulsive seizures as compared to saline-treated controls (Figure 8B; MPEP 25 mg/Kg, *p* < 0.05; MPEP 40 mg/Kg, *p* < 0.001; Kruskal-Wallis test followed by Dunn’s multiple comparison test). Overall, these data show that the mGluR5 antagonist MPEP attenuates epileptic seizure susceptibility in 3-week-old 3xTg-AD mice.

### MPEP Suppresses Prolonged Epileptiform Discharges in Hippocampal Slices from Young 3xTg-AD Mice

We next examined the effect of MPEP on prolonged epileptiform discharges elicited by bicuculline in hippocampal slices from young 3xTg-AD mice (Figures 8C,D). Prolonged epileptiform discharges were observed after extended (>30 min) exposure to bicuculline in 3xTg-AD hippocampal slices (Figures 8C,D; average duration of the five longest epileptiform discharges, bicuculline 20 min, 0.556 ± 0.096 s, vs. bicuculline 60 min, 5.259 ± 0.61 s, *n* = 9 slices from *n* = 5 mice; two-way ANOVA followed by Bonferroni *post hoc* test, *p* < 0.01). Within 30 min of MPEP (50 μM) perfusion, prolonged epileptiform discharges were no longer observed and only interictal epileptiform activity was recorded (Figures 8C,D; average duration of the five longest epileptiform discharges, bicuculline 60 min: 5.259 ± 0.610 s vs. bicuculline + MPEP 30 min: 0.526 ± 0.105 s, *n* = 9 slices from *n* = 5 mice; two-way ANOVA followed by Bonferroni *post hoc* test, *p* < 0.01). These results indicate that activation of mGluR5 sustains ictal-like activity in the hippocampal CA3 neuronal network of young 3xTg-AD mice.

## Discussion

The present study shows that as early as 3 weeks of age, 3xTg-AD mice exhibit high susceptibility to audiogenic seizures and exacerbated excitability of the hippocampal CA3 neuronal network. Our work provides evidence that network hyperexcitability precedes the onset of amyloid plaque and neurofibrillary pathologies, and memory impairment, and that intraneuronal human APP/Aβ expression and mGluR5 activation play a role in this process.

A few studies have previously assessed network hypersynchrony at young age in AD mouse models. Bezzina et al. (2015) recently reported the presence of spontaneous epileptiform activity (interictal spikes), increased susceptibility to phenylenetetrazole (PTZ, GABA_A_ receptor antagonist)-induced seizures, and ectopic expression of neuropeptide Y (NPY, marker of epileptic activity) in the mossy fibers of the hippocampus at 1.5 months of age (pre-amyloid plaque and pre-cognitive impairment stage) in Tg2576 mice which harbor hAPP_SWE_ mutation. Additionally, increased excitability in the entorhinal cortex of these mice has recently been shown at 2–4 months of age prior to extensive amyloid plaque deposition (Duffy et al., 2015); these mice were found to have soluble Aβ40 and Aβ42 in the entorhinal cortex as early as 2 months of age (Duffy et al., 2015). Another study showed the presence of interictal spikes on video EEG recordings in Tg2576 mice at 5 weeks of age (Kam et al., 2016). Increased susceptibility to PTZ-induced seizures was also reported in 6–8 week-old TgCRND8 mice (harboring hAPP_SWE_ and hAPP_Ind_ mutations) prior to amyloid plaque pathology and cognitive deficit (Del Vecchio et al., 2004). The presence of unprovoked electrographic seizures during 3-week recordings was also reported in the majority of 3- and 4.5-month-old APdE9 mice (harboring hAPP_SWE_ and PS1dE9 mutations) prior to amyloid plaque deposition and cognitive dysfunction (Minkeviciene et al., 2009). Thus, in recent years, the idea that hypersynchronous network activity precedes amyloid plaque pathology and cognitive dysfunction in AD mouse models has become widely recognized. However, the precise mechanism underlying this network hypersynchronization leading to precocious epileptic phenotype remains largely unknown.

In the 3xTg-AD mouse model, used in the current study, increased excitability in response to brief high-frequency activation in the hippocampal formation was reported at 4–6 months of age after development of episodic memory deficit (Davis et al., 2014). In addition, altered K_v_2.1 potassium channel function has recently been shown to mediate increased excitability in cultured hippocampal neurons (14–19 days *in vitro*) from embryonic day 16–18 triple transgenic mice (Frazzini et al., 2016). To the best of our knowledge, our data constitute the first clear demonstration of higher sensitivity to audiogenic seizures and hippocampal CA3 neuronal network hyperexcitability prior to onset of amyloid plaque deposition, neurofibrillary pathology, and cognitive deficit in this mouse line which carries human APP, tau and PS1 mutations.

Audiogenic seizures model human tonic-clonic seizures and epilepsy (Kandratavicius et al., 2014). Audiogenic seizures are mediated through brain auditory pathways with initiation in the midbrain inferior colliculus (Chakravarty and Faingold, 1999) and the hippocampus (Reid et al., 1983). Aβ pathology in AD is known to affect central auditory pathway including medial geniculate body and inferior colliculus nuclei (Sinha et al., 1993). Previously, increased sensitivity to audiogenic seizures was reported in 3-week-old Tg2576 (hAPP_SWE_) and Ts65Dn (APP triplication) mouse models of AD (Westmark et al., 2010). Our data show enhanced susceptibility to audiogenic seizures and reduced latency to the occurrence of convulsive seizures in 3-week-old 3xTg-AD mice. This seizure susceptibility in young 3xTg-AD mice was reduced by passive immunization with an anti-human APP/Aβ antibody or by mGluR5 blockade suggesting a role of intraneuronal human APP/Aβ prior to extracellular amyloid deposition and mGluR5 activation in exaggerated seizure susceptibility in these mice.

The present study shows evidence of enhanced excitability of the CA3 neuronal network in hippocampal slices from 3xTg-AD mice. Recurrent excitatory synaptic connections among CA3 neurons provide the cellular substrate for network synchronized firing (Traub and Wong, 1982; Cherubini and Miles, 2015) that underlie variant behavioral paradigms (Buzsáki et al., 1983) or, in conditions of suppressed inhibition, epileptiform activities (Traub and Wong, 1982, 1983a,b; Wong and Traub, 1983; Merlin and Wong, 1997; Bianchi et al., 2012). The hippocampal CA3 circuit is implicated in encoding spatial representations (O’Keefe and Nadel, 1978) and episodic memories (Scoville and Milner, 1957). The earliest neuropathological manifestations in AD are known to occur in the hippocampus and entorhinal cortex (Belleville et al., 2008; Reitz et al., 2009; Small et al., 2011). In mild cognitive impairment (MCI) subjects, CA3 neuronal hyperactivity has been reported, and dysfunctional encoding mechanisms in CA3 involved in episodic memory have been implicated (Bakker et al., 2012, 2015). The present study in 3-week-old 3xTg-AD mice is the first to document prolonged epileptiform discharges in CA3 pyramidal neurons in an AD mouse model. Given the critical role of CA3 auto-association network in episodic memory, this type of hypersynchronous network activity might be a contributory factor in hippocampal dysfunction leading to memory impairment in AD. In fact, recent studies provide strong evidence for the role of network hypersynchrony in cognitive impairment. The antiepileptic drug levetiracetam improved memory performance in MCI subjects (Bakker et al., 2012, 2015), and, in hAPPJ20 mice, suppressed epileptiform activity and ameliorated memory dysfunction (Sanchez et al., 2012). As shown in AD mice, early-onset hypersynchronous network activity may trigger changes in the hippocampal circuitry, including remodeling of inhibitory interneuron network, NPY ectopic expression, and probably aberrant neurogenesis, leading to progressive deterioration of hippocampal function that culminates in age-dependent cognitive decline (Palop et al., 2007; Palop and Mucke, 2009, 2016; Bezzina et al., 2015).

The early-onset epileptic activity in 3xTg-AD mice can be explained across multiple lines of evidence based on available literature and our data regarding the mechanism of this activity. The 3xTg-AD mice carry a mutated form of human APP resulting in intraneuronal human APP overexpression (3–4 fold), excessive Aβ peptide production, and its accumulation as amyloid plaques (Oddo et al., 2003a,b; Billings et al., 2005). Previous studies and our data show that 3xTg-AD mice are completely devoid of amyloid plaques at 3 weeks of age (Oddo et al., 2003a,b; Billings et al., 2005; Oh et al., 2010). This rules out the possibility that amyloid plaques cause early-onset epileptic activity in these mice. It was previously reported that the brains of 3-week-old 3xTg-AD mice contain intraneuronal human APP/Aβ (Oh et al., 2010), and our immunohistochemical analysis further confirmed it. Transgenic human APP itself has been suggested to lead to hypersynchronous network activity in an AD mouse model (Born et al., 2014). Additionally, Ts65Dn mice which carry three copies of the APP gene, also exhibit exaggerated susceptibility to audiogenic seizures as early as 3 weeks of age (Westmark et al., 2010). APP is a transmembrane protein which plays critical physiological roles in synapse formation and maturation (Dawkins and Small, 2014), and altered expression or processing may lead to network hypersynchronization. APP is cleaved by β-site APP cleaving enzyme 1 (BACE1). In AD transgenic mice that overexpress mutant APP, excess levels of this full-length APP may occupy most of BACE1 enzymatic activity, thus leading to a decrease in its capacity to process other substrates, including proteins that regulate ion channel function and ultimately neuronal excitability. For example, BACE1 cleaves the Navβ2 subunit of Nav1.1 channels, which regulates the functional α-subunit expression (Kim et al., 2007) in parvalbumin-expressing interneurons (Ogiwara et al., 2007). Alterations in Nav1.1 channels along with inhibitory interneuron dysfunction culminating in hypersynchronous network activity and memory impairment was reported in AD mouse models overexpressing APP/Aβ (Verret et al., 2012; Corbett et al., 2013). Other data show that loss of the potassium M-current in hippocampal neurons from BACE1 knockout mice enhances neuronal excitability and suggest that BACE1 physical association with the M-current KCNQ channels (Kv7) is required to sustain its regular function (Hessler et al., 2015). In the present study, we have not analyzed the membrane current(s) that may be altered in CA3 hippocampal neurons of young 3xTg-AD mice, however, consistent with the hypothesis of increased neuronal excitability resulting from human APP overexpression, our data indicate that intraneuronal human APP/Aβ leads to network hypersynchronization and ictal-like activity in this AD mouse model.

Our data are also consistent with intraneuronal overexpression of Aβ as a key factor causing enhanced hippocampal network hypersynchronization and seizure susceptibility in young 3xTg-AD mice. Intraneuronal Aβ accumulation was reported to induce hippocampal neuron hyperexcitability (Scala et al., 2015), which is known to cause early-onset cognitive dysfunction in 3xTg-AD mice (Billings et al., 2005). Also, APP Arctic mutation (E693G) transgenic mice with intraneuronal Aβ accumulation were reported to have increased susceptibility to PTZ-induced seizures (Ziyatdinova et al., 2016). We also cannot rule out the presence of soluble Aβ species and Aβ oligomers in 3-week-old 3xTg-AD mice, both of which were shown to induce neuronal hyperexcitability (Minkeviciene et al., 2009; Orbán et al., 2010; Zilberter et al., 2013). Soluble Aβ was reported to play a critical role in early-onset hippocampal hyperactivity in double transgenic (APP23 × PS45) AD mouse model (Busche et al., 2012). Overall, in the present study, because of their intrinsic relationship, we cannot exclusively separate out the impact of APP from that of Aβ in inducing early-onset network hypersynchronization in 3xTg-AD mice.

The present study shows that passive immunization with the anti-human APP/Aβ antibody, 6E10, significantly reduces intraneuronal transgenic APP/Aβ expression, which leads to a reduction in hippocampal CA3 hypersynchronous network activity and seizure susceptibility. The mouse monoclonal antibody, 6E10, recognizes N-terminal amino acids 1–16 of Aβ peptide. The passive immunization with 6E10 raises the possibility that 6E10 primarily acted on the N-terminus of Aβ in APP full-length and sAPPα on the cell surface and thereby decreased neuronal excitability. However, the present study also showed a significant reduction in human APP full-length and CTFs (APP-CT20) expression. These data indicate that passive immunization with 6E10 reduced intraneuronal human APP/Aβ. This seems plausible because, besides the N-terminal of Aβ peptide, 6E10 also recognizes CTFβ/C99 (Chang et al., 2003). Also, we found a positive correlation between intraneuronal human APP/Aβ expression, as quantified by either 6E10 or APP-CT20 antibody in CA3 neurons and ictal-like activity in the CA3 neuronal network.

Besides APP overexpression and Aβ pathology, the 3xTg-AD mice also exhibit tau overexpression, and tau hyperphosphorylation and aggregation (Oddo et al., 2003a,b). In the present study, the role of tau in early-onset network hyperexcitability was not evaluated and cannot be ruled out. It was shown that a transgenic mouse model overexpressing mutant human tau exhibits spontaneous epileptic activity and seizures with spike-wave complexes in the EEG recordings, and a higher sensitivity to PTZ (García-Cabrero et al., 2013). Additionally, reduction of endogenous tau was shown to ameliorate network hyperexcitability and cognitive impairment in transgenic APP mice (Roberson et al., 2007, 2011; Warmus et al., 2014; Hall et al., 2015). Conversely, a recent study showed that hyperphosphorylated tau reduces hippocampal CA1 neuronal hyperexcitability via relocation of the axon initial segment down the axon in P301L tau transgenic mice (Hatch et al., 2017). Thus, the role of tau in network hyperexcitability in AD warrants further in-depth evaluation.

Previous studies have shown that group I mGluRs, including mGluR5, are involved in the induction and maintenance of ictal-like activity in an *in vitro* model of epileptogenesis (Bianchi et al., 2012). Agonist stimulation of group I mGluRs was shown to elicit prolonged epileptiform (ictal-like) discharges in CA3 neurons of rodent WT hippocampal slices (Taylor et al., 1995; Merlin and Wong, 1997). However, synaptic activation of group I mGluRs—as it occurs during short bursts of glutamate release in slices treated with the GABA_A_ receptor antagonist bicuculline—is normally not sufficient to induce prolonged ictal-like discharges in WT slices. Induction of prolonged ictal-like discharges requires the activation of group I mGluR-dependent protein synthesis (Merlin et al., 1998). Sufficient synaptic activation of group I mGluR-dependent protein synthesis for induction of ictal-like activity is reached in preparations with gene knockout of translational repressor molecules (fragile X mental retardation protein, FMRP; BC1 RNA; Chuang et al., 2005; Zhong et al., 2009, 2010). After their induction, synaptic activation of group I mGluRs is also required to sustain ongoing prolonged ictal-like discharges (Lee et al., 2002; Chuang et al., 2005; Zhong et al., 2009, 2010; Young et al., 2013). The present study indicates that prolonged ictal-like discharges in the hippocampal CA3 neuronal network of 3-week-old 3xTg-AD mice were suppressed by mGluR5 blockade with MPEP, showing that synaptic activation of group I mGluRs was sufficient to sustain this ictal-like activity in the transgenic mice. Also, seizure susceptibility in young 3xTg-AD mice was also suppressed by MPEP. We noted that 3/35 (8.6%) of WT mice showed susceptibility to audiogenic seizures. We have not explored the underlying cellular mechanisms in WT. Our data indicate a role of mGluR5 in enhanced susceptibility to audiogenic seizures in 3xTg-AD mice. The results cannot determine whether this enhanced excitability is due to an exaggeration of a precondition to seizures in the WT or to the emergence of an mGluR-dependent epileptogenic process in the transgenic mice.

The data in the present study are consistent with mGluR-dependent hyperexcitability associated with APP overexpression. In the FXS mouse model (*Fmr1* knockout), absence of the translational repressor FMRP results in elevated APP levels (Westmark and Malter, 2007), and normalization of elevated APP levels by *App* knockdown reduces MPEP-sensitive ictal-like discharges in CA3 (Westmark et al., 2016) as well as audiogenic seizures (Westmark et al., 2011). The possible mechanism(s) by which APP/Aβ overexpression may increase mGluR-hyperexcitability were not directly investigated in the present study. As mentioned before, high levels of full-length APP may indirectly increase neuronal excitability by altering BACE1-dependent regulation of currents that are modulated by mGluRs. For example, the potassium M-current is decreased by reduced availability of BACE1 (Hessler et al., 2015) and is suppressed by mGluR activation (Charpak et al., 1990). These effects may be additive in the transgenic mice resulting in larger depolarization and prolonged firing. Another study shows that intracellular APP/Aβ overexpression results in extracellular levels of soluble Aβ oligomers (Aβo) that are sufficient to bind to the cellular prion protein (PrP^C^) and activate mGluR5 (Um et al., 2013). Such binding has been shown to induce inward currents in transfected oocytes and intracellular calcium increase in cultured cortical neurons (Um et al., 2013).

In summary, the present study shows that seizure susceptibility and hypersynchronous network activity precedes amyloid plaque pathology and memory dysfunction in 3xTg-AD mice. This early-onset network hyperexcitability can be suppressed by passive immunization with an anti-human APP/Aβ antibody and mGluR5 antagonist, MPEP. The early-onset network hyperexcitability can culminate in progressive modifications in hippocampal circuitry, ultimately contributing to memory impairment in AD mice. In humans, hypersynchronous network activity could potentially be used as a biomarker to predict memory decline. Additionally, the present study provides rationale for employing Aβ passive immunization at early human AD stages to exert maximal beneficial effect. The mGluR5 antagonists need to be further evaluated for their beneficial effect on network hyperexcitability, and potentially on cognitive dysfunction in AD.

## Author Contributions

SFK performed most of the experimental work including audiogenic seizures, electrophysiology, immunohistochemistry, analyzed the data and wrote the manuscript. S-CC assisted in electrophysiology experiments and data analysis. WZ helped in audiogenic seizure experiments and data analysis. RKSW guided the electrophysiology and audiogenic seizure experiments, data presentation and wrote the manuscript. RB and KI provided overall supervision for the study, and conceived and directed all phases of the study, including the manuscript. All authors read and approved the final manuscript.

## Conflict of Interest Statement

The authors declare that the research was conducted in the absence of any commercial or financial relationships that could be construed as a potential conflict of interest.
